# Coffee, Alcohol, Smoking, Physical Activity and QT Interval Duration: Results from the Third National Health and Nutrition Examination Survey

**DOI:** 10.1371/journal.pone.0017584

**Published:** 2011-02-28

**Authors:** Yiyi Zhang, Wendy S. Post, Darshan Dalal, Elena Blasco-Colmenares, Gordon F. Tomaselli, Eliseo Guallar

**Affiliations:** 1 Department of Epidemiology, Johns Hopkins University Bloomberg School of Public Health, Baltimore, Maryland, United States of America; 2 Welch Center for Prevention, Epidemiology, and Clinical Research, Johns Hopkins Medical Institutions, Baltimore, Maryland, United States of America; 3 Division of Cardiology, Department of Medicine, Johns Hopkins University School of Medicine, Baltimore, Maryland, United States of America; 4 Department of Cardiovascular Epidemiology and Population Genetics, National Center for Cardiovascular Research (CNIC), Madrid, Spain; Lerner Research Institute, Cleveland Clinic, United States of America

## Abstract

**Background:**

Abnormalities in the electrocardiographic QT interval duration have been associated with an increased risk of ventricular arrhythmias and sudden cardiac death. However, there is substantial uncertainty about the effect of modifiable factors such as coffee intake, cigarette smoking, alcohol consumption, and physical activity on QT interval duration.

**Methods:**

We studied 7795 men and women from the Third National Health and Nutrition Survey (NHANES III, 1988–1994). Baseline QT interval was measured from the standard 12-lead electrocardiogram. Coffee and tea intake, alcohol consumption, leisure-time physical activities over the past month, and lifetime smoking habits were determined using validated questionnaires during the home interview.

**Results:**

In the fully adjusted model, the average differences in QT interval comparing participants drinking ≥6 cups/day to those who did not drink any were −1.2 ms (95% CI −4.4 to 2.0) for coffee, and −2.0 ms (−11.2 to 7.3) for tea, respectively. The average differences in QT interval duration comparing current to never smokers was 1.2 ms (−0.6 to 2.9) while the average difference in QT interval duration comparing participants drinking ≥7 drinks/week to non-drinkers was 1.8 ms (−0.5 to 4.0). The age, race/ethnicity, and RR-interval adjusted differences in average QT interval duration comparing men with binge drinking episodes to non-drinkers or drinkers without binge drinking were 2.8 ms (0.4 to 5.3) and 4.0 ms (1.6 to 6.4), respectively. The corresponding differences in women were 1.1 (−2.9 to 5.2) and 1.7 ms (−2.3 to 5.7). Finally, the average differences in QT interval comparing the highest vs. the lowest categories of total physical activity was −0.8 ms (−3.0 to 1.4).

**Conclusion:**

Binge drinking was associated with longer QT interval in men but not in women. QT interval duration was not associated with other modifiable factors including coffee and tea intake, smoking, and physical activity.

## Introduction

Extremely abnormal prolongation or reduction of the electrocardiographic QT interval duration, such as seen in individuals with Mendelian forms of long or short QT syndromes, is associated with increased risk of ventricular arrhythmias and sudden cardiac death [1]–[5]. Furthermore, population studies have also shown associations between smaller increases in QT interval duration and total mortality, cardiovascular mortality, and sudden cardiac death [6]–[17].

Besides genetic disorders and pharmacologic agents that can cause marked prolongation or shortening of the QT interval [18], other factors associated with less extreme QT interval variability in the general population include age, sex, hypertension, body mass index, low-calorie diets, electrolytes [19]–[20], and common genetic variants [21]. However, there is substantial uncertainty about the association of modifiable factors, such as coffee intake, cigarette smoking, alcohol consumption, and physical activity with QT interval duration [22]–[34]. Although several studies have assessed the effect of individual risk factors on QT interval, many of them were small and/or based on selected samples, resulting in inconsistent findings. The purpose of this analysis was thus to investigate the association of coffee, tea, or alcohol intake, smoking, and physical activity with QT interval duration in a large representative sample of the general US population.

The NHANES III study was conducted by the National Center for Health Statistics (NCHS) of the Centers for Disease Control and Prevention (CDC). It is a national survey that collects extensive health information of the general US population from both interviews and medical examinations. The NHANES III study was approved by the NCHS Research Ethics Review Board (ERB), and documented consent was obtained from participants.

## Methods

### Study Population

We analyzed data from the Third National Health and Nutrition Examination Survey (NHANES III), a cross-sectional study conducted between 1988 and 1994 that used a multistage stratified clustered probability design to select a representative sample of the civilian non-institutionalized US population [35]. The present study was restricted to participants 40 years of age and older as 12-lead electrocardiograms (ECGs) were only performed in this age group. Of the 8,561 participants 40 years of age or older who had available ECG measurements, we excluded 194 participants with missing QT interval duration or heart rate, 535 participants with QRS ≥120 ms, and 37 participants with missing data on coffee, tea, or alcohol intake, smoking, or physical activity. The final analysis was based on 7,795 participants (3,682 men and 4,113 women).

### Data collection

NHANES III included a standardized questionnaire administered in the home by a trained interviewer and a detailed physical examination at a mobile examination center. Demographics, education, medical history, and medication use were assessed by interview. QT-prolonging medications were defined according to the Arizona Center for Education and Research on Therapeutics database [36]. Height and weight were measured and body mass index was calculated as weight in kilograms divided by height in meters squared. Blood pressure was measured three times during the in-home interview and three additional times during the participant’s visit to the mobile examination center. Laboratory tests included total cholesterol, HDL cholesterol, plasma glucose, serum potassium, and serum calcium. Diabetes was defined as a fasting plasma glucose ≥126 mg/dL, a nonfasting plasma glucose ≥200 mg/dL, and/or current use of oral hypoglycemic agents or insulin.

### Exposure assessment

Coffee and tea consumption over the past month were determined using a food frequency questionnaire during the home interview [35]. The questionnaire asked how often the participants had regular coffee or tea per month, and we categorized the responses into 0, <1, 1–3, 4–5, and ≥6 cups/day. Based on the U.S. Department of Agriculture food composition sources, we estimated that the caffeine of a cup of coffee or tea were 137 and 47 mg, respectively [37]–[38], and used the frequency of coffee and tea consumption to estimate caffeine intake. In NHANES III, regular colas and sodas were coded together in one variable. Since not all sodas contain caffeine, we did not include colas when calculating total caffeine intake in the main analyses, but performed sensitivity analyses including the contribution of regular colas and sodas to caffeine, assuming that one drink contained 46 mg of caffeine. Caffeine intake was categorized into quartiles in the analysis.

Participants were asked if they had smoked more than 100 cigarettes in their lifetime and if they were current smokers during the home interview. In addition, serum cotinine, a valid and reliable biomarker of exposure to tobacco smoke, was analyzed using high performance liquid chromatography/atmospheric-pressure ionization tandem mass spectrometry [39]. In the present analysis, current smokers were defined as those who self-reported as current smokers, or those who had serum cotinine >10 ng/ml [40]. Former smokers were those participants who had smoked more than 100 cigarettes but were not current smokers. Participants who had not smoked more than 100 cigarettes in their lifetime and had serum cotinine <10 ng/ml were considered never smokers. Current smokers were further categorized into quartiles based on pack-years smoked (a pack-year defined as 20 cigarettes/day for 1 year), and serum cotinine concentrations [41]. In addition, never smokers who were exposed to second-hand smoke (SHS exposed never smokers) were further separated from never smokers who were not exposed to passive smoking (SHS unexposed never smokers). Since the detection limit of continine in NHANES III was 0.05 ng/ml, SHS unexposed never smokers were defined as never smokers with cotinine level <0.05 ng/ml, while SHS exposed nonsmoker were defined as never smokers with cotinine level between 0.05 and 10 ng/ml [42].

Alcohol consumption was also assessed by the food frequency questionnaire. Participants reported the number of times that they drank beer, wine, and hard liquor in the past month and we categorized total alcohol consumption into 4 groups (0, 1–3, 4–6, ≥7 drinks/week). In addition, the frequency of heavy episodic drinking was assessed during the alcohol and drug component of the examination. Participants were classified as binge drinkers if they had at least five alcoholic drinks in a single day during the past 12 months.

Leisure-time physical activity in the past month was ascertained during the home interview. Physical activities were coded and classified according to the rate of energy expenditure using a standardized scheme [43]. Moderate physical activities included walking, biking, swimming, aerobics, dancing, calisthenics, gardening, lifting weights, and other physical activities if they met age-specific cut-offs of metabolic equivalents (METs): ≥3.0 METs for ages 20–39 years; ≥2.5 METs for ages 40–64 years; ≥2.0 METs for ages 65–79 years; and ≥1.26 METs for age 80 years or older. Vigorous physical activity included jogging or running. In addition, swimming and aerobics were classified as vigorous for participants 40 years or older; biking, dancing, gardening, and calisthenics were classified as vigorous for participants 65 years or older; and walking and lifting weights were classified as vigorous for participants 80 years and older. Other physical activities were also considered vigorous if they met age-specific MET cut-offs: ≥7.2 METs for ages 20–39 years, ≥6.0 METs for ages 40–64 years, ≥4.8 METs for ages 65–79 years, and ≥3.0 METs for age 80 years and older [41]. Total (moderate or vigorous) physical activity was categorized into 0, <3, 3–7.9, and ≥8 times/week. Vigorous physical activity was categorized into 0, <1, 1–4, and >4 times/week.

### QT interval

Standard 12-lead resting ECG recordings were performed using a Marquette MAC 12 electrocardiograph (Marquette Medical Systems, Inc., Milwaukee, WI, USA) with signals sampled at 250 samples per second per channel. A representative P-QRS-T cycle was then derived by selective averaging using the Dalhousie ECG Analysis Program [44]. Resting heart rates and QT intervals were obtained from the ECGs. All ECGs were read centrally at the Epidemiological Cardiology Research Center, EPICARE (Wake Forest University, Winston-Salem, NC).

### Statistical Analysis

The ECG sampling weights were used in the analysis to account for the complex sampling design [35]. The primary effect measure was the adjusted difference and 95% confidence interval (CI) in QT interval duration by categories of each exposure compared to the reference category, calculated from multivariable linear regression models. We used four models with progressive degrees of adjustment. First, we adjusted for age (continuous), race-ethnicity (non-Hispanic white, non-Hispanic black, Mexican-American, other), sex, and RR-interval (restricted quadratic splines with knots at the 5^th^, 50^th^, and 95^th^ percentiles). Second, we further adjusted for BMI (continuous), high school education (yes, no), annual household income (<$20,000, ≥$20,000), and use of QT-prolonging medications (yes, no). Third, we further adjusted for systolic blood pressure, blood pressure lowering medication, total and HDL cholesterol, diabetes, history of myocardial infarction, history of congestive heart failure, serum potassium (continuous), and serum calcium (continuous). Finally, fully adjusted models further included all exposures of interest (i.e., coffee, tea, and alcohol intake, smoking, and physical activity) in a single model. Tests for linear trend across categories of each exposure were computed by including a variable with the median value for each category of exposure in the linear regression models.

Seconday analyses included stratified analysis by sex (as women tend to have longer QT intervals than men), and sensitivity analysis using Bazett’s corrected QT interval as the outcome (Table S1; results were similar to the main analysis). All statistical analyses were conducted using SUDAAN (version 10.0; Research Triangle Institute, Research Triangle Park, NC).

## Results

The average age of study participants was 56.5 years, and 45.1% of them were male (Table 1). Non-Hispanic whites comprised 80.7% of the study population. The average duration of the QT interval was 406.3 ms.

**Table 1:** Baseline characteristics.

Characteristic	NHANES III (N = 7,795)
Age (years) ^a^	56.5 (0.4)
Male	45.1
Race/ethnicity	
White	80.7
Black	9.0
Other	10.4
High school education	70.8
Low family income	30.9
Use of QT prolonging medication	12.0
Diabetes	8.6
Myocardial infarction	5.2
Hypertension	36.7
Total cholesterol (mg/dL)	218.0 (0.9)
HDL (mg/dL)	51.0 (0.4)
BMI (kg/m^2^)	27.3 (0.1)
Heart rate (beat/min)	68.1 (0.2)
QT interval (ms)	406.3 (0.6)

Values are means (SE) or percentages unless otherwise noted.

In the fully adjusted model, the average differences in QT interval comparing participants drinking ≥6 cups/day to those who did not drink any were −1.2 (95% CI −4.4 to 2.0) for coffee, and −2.0 ms (−11.2 to 7.3) for tea, respectively (Table 2). The average difference in QT interval comparing the highest vs. the lowest quartiles of caffeine intake was −1.2 ms (−2.7 to 0.3). With respect to smoking, the average differences in QT interval duration comparing current and former smokers to never smokers in fully adjusted models were 1.2 ms (−0.6 to 2.9) and 0.4 ms (−0.9, 1.7), respectively (Table 3). Among smokers, the average difference in QT interval comparing the highest vs. the lowest quartiles of pack-years was 2.6 ms (−1.0 to 6.2), while the average difference comparing highest vs. the lowest quartiles of serum cotinine concentrations was 1.5 ms (−2.1 to 5.2). SHS exposed never smokers had similar QT interval durations as SHS unexposed never smokers.

**Table 2:** Adjusted difference (95%CI) in QT interval duration by categories of coffee, tea, and caffeine intake.

	N	Mean QT	Model 1 a	Model 2 b	Model 3 c	Model 4 d
**Coffee (cups/day)**						
0	2347	406.8 (404.8, 408.9)	0.0 (reference)	0.0 (reference)	0.0 (reference)	0.0 (reference)
<1	3671	406.7 (405.2, 408.3)	−0.7 (−2.0, 0.5)	−0.8 (−2.1, 0.5)	−0.7 (−1.9, 0.5)	−0.9 (−2.2, 0.4)
1–3	1257	405.0 (402.7, 407.4)	−1.8 (−3.2, −0.3)	−1.5 (−3.0, 0.0)	−1.2 (−2.7, 0.3)	−1.6 (−3.2, 0.1)
4–5	311	402.0 (399.0, 405.1)	−0.2 (−2.7, 2.3)	0.5 (−1.9, 3.0)	1.1 (−1.3, 3.5)	0.6 (−2.0, 3.2)
≥6	209	404.7 (400.1, 409.2)	−1.1 (−4.3, 2.2)	−1.1 (−4.5, 2.4)	−0.7 (−4.1, 2.8)	−1.2 (−4.4, 2.0)
p-trend		0.03	0.42	0.61	0.89	0.59
**Tea (cups/day)**						
0	4137	407.2 (405.6, 408.7)	0.0 (reference)	0.0 (reference)	0.0 (reference)	0.0 (reference)
<1	3242	405.3 (403.8, 406.8)	−0.4 (−1.5, 0.8)	−0.4 (−1.6, 0.7)	−0.2 (−1.4, 1.0)	−0.1 (−1.4, 1.1)
1–3	343	403.4 (398.0, 408.8)	−1.8 (−4.5, 1.0)	−2.3 (−5.1, 0.5)	−2.4 (−5.4, 0.5)	−2.4 (−5.3, 0.6)
4–5	58	403.8 (393.3, 414.3)	−0.1 (−4.9, 4.7)	−0.8 (−5.2, 3.6)	0.8 (−4.2, 5.8)	0.6 (−4.3, 5.6)
≥6	15	405.9 (393.7, 418.2)	0.3 (−9.1, 9.7)	1.2 (−8.4, 10.7)	−2.1 (−11.4, 7.2)	−2.0 (−11.2, 7.3)
p-trend		0.18	0.44	0.33	0.27	0.27
**Caffeine (mg/day)**						
<24.2	2080	408.4 (406.3, 410.4)	0.0 (reference)	0.0 (reference)	0.0 (reference)	0.0 (reference)
24.2–135.2	2425	405.8 (404.2, 407.4)	−1.6 (−3.0, −0.1)	−1.6 (−3.1, −0.2)	−1.1 (−2.6, 0.4)	−1.3 (−2.8, 0.3)
135.2–274.9	1489	406.6 (404.0, 409.3)	−0.1 (−1.4, 1.3)	−0.1 (−1.6, 1.3)	0.1 (−1.4, 1.6)	−0.1 (−1.6, 1.5)
≥274.9	1801	404.2 (402.5, 405.8)	−1.6 (−2.9, −0.2)	−1.3 (−2.7, 0.1)	−0.8 (−2.2, 0.6)	−1.2 (−2.7, 0.3)
p-trend		<0.001	0.05	0.13	0.35	0.17

a. Adjusted for age (continuous), race-ethnicity (non-Hispanic white, non-Hispanic black, Mexican-American, other), sex, and RR-interval (restricted quadratic splines with knots at the 5th, 50th, and 95th percentiles).

b. Further adjusted for BMI (continuous), high school education (yes, no), annual household income (<$20,000, ≥$20,000), and use of QT-prolonging medications (yes, no).

c. Further adjusted for systolic blood pressure, blood pressure lowering medication, total and HDL cholesterol, diabetes, history of myocardial infarction, history of congestive heart failure, serum potassium (continuous), and serum calcium (continuous).

d. Further adjusted for smoking (current, former, never), number of drinks (continuous), and total physical activity (continuous).

**Table 3:** Adjusted difference (95%CI) in QT interval duration by categories of smoking, pack-years, and serum cotinine.

	N	Mean QT	Model 1 a	Model 2 b	Model 3 c	Model 4 d
**Smoking**						
Never	3306	407.8 (406.2, 409.4)	0.0 (reference)	0.0 (reference)	0.0 (reference)	0.0 (reference)
Former	2247	407.6 (405.7, 409.6)	0.5 (−0.9, 1.9)	0.4 (−1.0, 1.8)	0.4 (−0.9, 1.7)	0.4 (−0.9, 1.7)
Current	2242	402.1 (400.0, 404.3)	0.7 (−1.0, 2.4)	0.9 (−0.9, 2.6)	1.2 (−0.5, 2.9)	1.2 (−0.6, 2.9)
p-value (former vs. never)		0.90	0.49	0.56	0.54	0.57
p-value (current vs. never)		<0.001	0.45	0.34	0.16	0.20
**Pack-years (current smokers only)**						
≤14.9	697	405.3 (402.2, 408.3)	0.0 (reference)	0.0 (reference)	0.0 (reference)	0.0 (reference)
14.9–31.4	588	400.9 (396.9, 404.8)	0.5 (−2.6, 3.6)	0.2 (−3.0, 3.3)	0.4 (−2.7, 3.5)	0.3 (−2.8, 3.4)
31.4–49.7	447	400.0 (395.5, 404.5)	0.7 (−2.2, 3.7)	0.9 (−2.1, 3.9)	0.8 (−2.6, 4.2)	0.7 (−2.8, 4.1)
≥49.7	437	402.0 (397.8, 406.3)	3.1 (0.0, 6.2)	3.0 (−0.2, 6.1)	2.8 (−0.7, 6.3)	2.6 (−1.0, 6.2)
p-trend		0.23	0.04	0.05	0.11	0.15
**Serum cotinine (ng/ml, current smokers only)**						
<127.7	623	406.0 (403.1, 408.9)	0.0 (reference)	0.0 (reference)	0.0 (reference)	0.0 (reference)
127.7–226.8	493	404.3 (399.1, 409.6)	2.4 (−0.8, 5.6)	2.5 (−0.8, 5.8)	2.8 (−0.8, 6.4)	2.7 (−0.9, 6.3)
226.8–327.6	494	398.8 (394.6, 403.0)	−1.1 (−4.0, 1.7)	−0.8 (−3.8, 2.3)	−0.4 (−3.6, 2.8)	−0.5 (−3.8, 2.7)
>327.6	553	399.0 (395.1, 403.0)	0.8 (−2.5, 4.1)	0.9 (−2.3, 4.2)	1.7 (−1.9, 5.2)	1.5 (−2.1, 5.2)
p-trend		0.00	0.99	0.89	0.63	0.69
**Secondhand smoking (SHS)**						
SHS unexposed never smokers	688	410.5 (406.1, 414.9)	0.0 (reference)	0.0 (reference)	0.0 (reference)	0.0 (reference)
SHS exposed never smokers	2418	407.1 (405.6, 408.6)	0.5 (−2.4, 3.3)	0.2 (−2.6, 2.9)	0.9 (−2.0, 3.8)	0.9 (−2.0, 3.8)
Current smokers	2242	402.1 (400.0, 404.3)	1.1 (−1.8, 4.1)	1.1 (−1.8, 4.0)	2.2 (−0.9, 5.2)	2.0 (−1.0, 5.1)
p-value (SHS exposed vs.SHS unexposed never smokers)		0.12	0.74	0.91	0.56	0.55
p-value (Current smokers vs. SHS unexposed never smoker)		<0.001	0.45	0.46	0.17	0.20

a. Adjusted for age (continuous), race-ethnicity (non-Hispanic white, non-Hispanic black, Mexican-American, other), sex, and RR-interval (restricted quadratic splines with knots at the 5th, 50th, and 95th percentiles).

b. Further adjusted for BMI (continuous), high school education (yes, no), annual household income (<$20,000, ≥$20,000), and use of QT-prolonging medications (yes, no).

c. Further adjusted for systolic blood pressure, blood pressure lowering medication, total and HDL cholesterol, diabetes, history of myocardial infarction, history of congestive heart failure, serum potassium (continuous), and serum calcium (continuous).

d. Further adjusted for coffee (continuous), tea (continuous), number of drinks (continuous), and total physical activity (continuous).

The average difference in QT interval duration comparing participants drinking ≥7 drinks/week to non-drinkers in fully adjusted models was 1.8 ms (−0.5 to 4.0) (Table 4). After adjusting for age, race/ethnicity, sex and RR-interval, the average differences in QT interval duration comparing binge drinking to non-drinkers or to drinkers without binge drinking were 2.2 (0.1 to 4.4) and 3.1 ms (0.8 to 5.3), respectively. In the sex-stratified analysis, the average differences in QT interval comparing binge drinking to non-drinkers or drinkers without binge drinking were 2.8 (0.4 to 5.3) and 4.0 ms (1.6 to 6.4), respectively, among men, and 1.1 ms (−2.9 to 5.2) and 1.7 ms (−2.3 to 5.7), respectively, among women (Table S2). The interaction term for sex and binge drinking was not significant (p-value 0.34), although this analysis was limited by the relatively small number of women with binge drinking (282 women vs. 1005 men).

**Table 4:** Adjusted difference (95%CI) in QT interval duration by categories of alcohol consumption.

	N	Mean QT	Model 1 a	Model 2 b	Model 3 c	Model 4 d ^,^ e
**Alcohol (drinks/week)**						
0	4513	406.0 (404.5, 407.5)	0.0 (reference)	0.0 (reference)	0.0 (reference)	0.0 (reference)
1–3	2143	405.7 (404.0, 407.4)	−0.3 (−1.9, 1.2)	−0.2 (−1.8, 1.4)	−0.4 (−2.0, 1.3)	−0.5 (−2.1, 1.2)
4–6	340	406.8 (402.5, 411.0)	0.0 (−2.9, 2.9)	0.6 (−2.3, 3.6)	0.3 (−2.6, 3.1)	0.2 (−2.7, 3.1)
≥7	799	407.4 (403.2, 411.6)	1.7 (−0.5, 3.8)	2.7 (0.4, 4.9)	1.9 (−0.3, 4.1)	1.8 (−0.5, 4.0)
p-trend		0.45	0.14	0.02	0.09	0.11
**Binge drinking**						
Non-drinker or ex-drinker	4623	406.0 (404.4, 407.6)	0.0 (reference)	0.0 (reference)	0.0 (reference)	0.0 (reference)
Current drinker, binge drinking	1287	403.5 (400.6, 406.4)	2.2 (0.1, 4.4) f	2.4 (0.3, 4.5) f	1.9 (−0.1, 3.9) f	1.6 (−0.6, 3.7) f
p-value f		0.14	0.05	0.03	0.07	0.16
Current drinker, no binge drinking	1631	408.2 (406.2, 410.1)	0.0 (reference)	0.0 (reference)	0.0 (reference)	0.0 (reference)
Current drinker, binge drinking	1287	403.5 (400.6, 406.4)	3.1 (0.8, 5.3) g	2.7 (0.5, 5.0) g	2.2 (−0.1, 4.6) g	2.1 (−0.4, 4.5) g
p-value g		0.01	0.01	0.02	0.07	0.10

a. Adjusted for age (continuous), race-ethnicity (non-Hispanic white, non-Hispanic black, Mexican-American, other), sex, and RR-interval (restricted quadratic splines with knots at the 5th, 50th, and 95th percentiles).

b. Further adjusted for BMI (continuous), high school education (yes, no), annual household income (<$20,000, ≥$20,000), and use of QT-prolonging medications (yes, no).

c. Further adjusted for systolic blood pressure, blood pressure lowering medication, total and HDL cholesterol, diabetes, history of myocardial infarction, history of congestive heart failure, serum potassium (continuous), and serum calcium (continuous).

d. Further adjusted for coffee (continuous), tea (continuous), smoking (current, former, never), and total physical activity (continuous).

e. Model 4 for binge drinking further adjusted for number of drinks (continuous).

f. Current drinker, binge drinking vs. non-drinker or ex- drinker.

g. Current drinker, binge drinking vs. current drinker, no binge drinking.

Finally, the average differences in QT interval comparing the highest vs. the lowest categories of total and vigorous physical activity in the fully adjusted model were −0.8 (−3.0 to 1.4) and −0.3 ms (−2.9 to 2.4), respectively (Table 5). When stratified by sex, there was a trend towards shorter QT intervals with increasing levels of total activity in women but not in men in the fully adjusted model (p-trend 0.83 in men and 0.03 in women; p-interaction 0.09, Table S2).

**Table 5:** Adjusted difference (95%CI) in QT interval duration by categories of physical activity.

	N	Mean QT	Model 1 a	Model 2 b	Model 3 c	Model 4 d
**Total physical activity (times/week)**						
0	2540	402.8 (400.3, 405.2)	0.0 (reference)	0.0 (reference)	0.0 (reference)	0.0 (reference)
0.1–2.9	2918	405.1 (403.6, 406.6)	−0.3 (−2.1, 1.5)	0.2 (−1.6, 2.0)	0.4 (−1.3, 2.2)	0.5 (−1.3, 2.2)
3.0–7.9	1524	408.7 (406.8, 410.6)	−1.0 (−3.1, 1.0)	−0.4 (−2.4, 1.6)	−0.7 (−2.7, 1.2)	−0.7 (−2.7, 1.3)
≥8.0	813	410.6 (407.4, 413.8)	−2.2 (−4.1, −0.2)	−1.0 (−3.0, 1.0)	−0.9 (−3.0, 1.3)	−0.8 (−3.0, 1.4)
p-trend		<0.001	0.03	0.23	0.20	0.20
**Vigorous physical activity (times/week)**						
0	5103	403.4 (402.1, 404.8)	0.0 (reference)	0.0 (reference)	0.0 (reference)	0.0 (reference)
0.1–1.0	881	405.8 (403.3, 408.4)	−1.6 (−3.3, 0.1)	−1.1 (−2.9, 0.7)	−0.6 (−2.5, 1.3)	−0.6 (−2.5, 1.3)
1.1–4.0	819	411.2 (408.5, 413.8)	−0.7 (−2.7, 1.3)	−0.3 (−2.3, 1.8)	−0.3 (−2.5, 1.9)	−0.2 (−2.5, 2.0)
>4	992	414.7 (410.9, 418.5)	−0.9 (−3.1, 1.4)	0.0 (−2.4, 2.3)	−0.3 (−2.9, 2.3)	−0.3 (−2.9, 2.4)
p-trend		<0.001	0.52	0.95	0.83	0.88

a. Adjusted for age (continuous), race-ethnicity (non-Hispanic white, non-Hispanic black, Mexican-American, other), sex, and RR-interval (restricted quadratic splines with knots at the 5th, 50th, and 95th percentiles).

b. Further adjusted for BMI (continuous), high school education (yes, no), annual household income (<$20,000, ≥$20,000), and use of QT-prolonging medications (yes, no).

c. Further adjusted for systolic blood pressure, blood pressure lowering medication, total and HDL cholesterol, diabetes, history of myocardial infarction, history of congestive heart failure, serum potassium (continuous), and serum calcium (continuous).

d. Further adjusted for coffee (continuous), tea (continuous), smoking (current, former, never), and number of drinks (continuous).

## Discussion

In a large sample representative of the general US population, we found no association between QT interval duration and coffee or tea intake, cigarette smoking, physical activity, or total alcohol intake, although binge drinking was associated with an increased QT duration particularly in men.

### Coffee, tea, and caffeine

Few studies have evaluated the effect of coffee or tea intake on the QT interval, although a few small studies have assessed the short-term effect of caffeine on cardiac repolarization. A study of 18 healthy subjects and 18 patients with frequent ventricular ectopic beats reported no significant change in QT interval in either group after caffeine ingestion (multiple doses of 1 mg/kg of body weight at intervals of one half-life during waking hours) [34]. Another study of 10 healthy volunteers showed that caffeine consumption (400 mg/day, equivalent of 4 cups of coffee) did not affect the QT interval [22]. In addition, experiments with canine ventricular muscle models found no effect of caffeine on cardiac action potentials, which may explain its lack of effect on the QT interval [45]. Our results were consistent with previous research and further suggested that usual intake of caffeine-containing drinks does not affect QT interval duration.

### Cigarette smoking

Previous studies have shown conflicting results regarding the influence of acute and chronic smoking on QT interval duration. Some studies have reported longer QT intervals in smokers compared to non-smokers [25], [27]–[28], [46], while others found no significant differences [47] or even shorter QT intervals [29]. Another study showed that smoking cessation reduced the QT interval [32]. Most of these studies had small sample sizes and used only univariate analysis without adjustment for confounders. Furthermore, in some studies the results were dependent on the choice of formula for heart rate correction [23]. Our analysis, with a much larger sample size and more detailed adjustment for potential confounders, suggest a lack of effect of chronic smoking on QT interval duration, although we cannot completely exclude an association between smoking history, reflected in pack-years of smoking, and an increase in QT interval duration.

### Alcohol consumption

A substantial body of literature has identified a variety of ECG abnormalities, including prolonged QT interval, in chronic alcoholics or heavy drinkers. Abnormalities of electrolytes (hypomagnesemia, hypopotassemia) [48]–[50], increased sympathetic tone and catecholamine secretion [51], as well as cardiac cellular infiltrate, hypertrophy, and fibrosis [52]–[53] are frequently seen in chronic alcoholism, which may all cause changes in QT interval. Electromechanical experiments also suggested a concentration-dependent effect of alcohol on action potential duration, with a decreased duration of repolarization at very high concentrations of alcohol but no effect at low concentrations [54]. Several studies of chronic alcoholics have reported either high incidences of prolonged QT (QTc>440 ms) [30]–[31], or longer QT intervals compared to normal controls [24], [26]. In addition, prolongation of the QT interval has also been reported after acute alcohol infusion [33]. In the present analysis, binge drinking, defined as five or more alcoholic drinks in one day during the past 12 months, may identify individuals with excessive heavy alcohol use and who were more likely to have chronic alcoholism. Our results suggested that binge drinking may prolong the QT interval compared to non-drinkers or drinkers without binge drinking, particularly in men. Compared to chronic alcoholism, less is known about the effect of social drinking on QT interval duration in the general population. The only population-based study we could identify (2,894 healthy men and women) showed no association between alcohol intake and QT duration [27]. Our data confirmed this observation that usual alcohol consumption was not associated with QT interval duration.

### Physical activity

We did not find an association between physical activity and QT interval duration. When stratified by sex, the results suggested a trend towards shorter QT intervals with increasing levels of total activity in women but not in men. However, the test for interaction between sex and total physical activity was not significant, and a similar trend was not observed for vigorous physical activity. A previous study reported that high physical activity was associated with an increase in QT interval in men but not women [27]. It was hypothesized that a higher left ventricular mass could explain this association [27], and that this effect may only be observed at very high levels of physical activity. Differences in study population, levels of physical activity, and measurement of physical activity may explain the discrepancy with our findings.

Several limitations of our study need to be considered. First, all exposure factors and QT interval duration were measured at a single time at baseline, which may result in non-differential measurement error as there is substantial within person variability in all these variables. It is thus possible that our analysis may have missed some small associations between exposure factors and QT interval duration. Second, coffee, tea, alcohol, smoking and physical activity are associated with many other behaviors and cardiovascular risk factors, and we cannot exclude residual confounding. Third, the currenly analysis could not differentiate between the acute and long-term effect of each exposure factor due to the limited information available regarding duration and magnitude of exposure. Finally, QT interval duration is an intermediate physiological variable, and the impact of the observed changes on clinical cardiovascular events is uncertain. Some major strengths of this study are its large sample size, the careful standardization and detailed quality control procedures of NHANES, and the generalizability of the findings to the general US population.

In conclusion, data from NHANES III, a large sample representative of the general US population, found no association between QT interval duration and coffee or tea intake, smoking, physical activity and usual drinking, but suggested an association between binge drinking and longer QT interval in men. Future studies are needed to further elucidate the biological mechanisms underlying the observed association between binge drinking and QT interval duration and the role of heavy alcohol intake in QT abnormalities and arrhythmia triggering in the general population.

## Supporting Information

Table S1 Adjusted difference (95%CI) in Bazett’s equation-corrected QT (QTb) interval duration.
(DOC)

Table S2 Adjusted difference (95%CI) in QT interval by sex.
(DOC)
