# Supplementary material for: Coffee, Alcohol, Smoking, Physical Activity and QT Interval Duration: Results from the Third National Health and Nutrition Examination Survey
Source: PLoS One. 2011 Feb 28;6(2):e17584. doi: 10.1371/journal.pone.0017584 (PMC3046251; doi:10.1371/journal.pone.0017584)
Supplement: Table S1 — Adjusted difference (95%CI) in Bazett’s equation-corrected QT (QTb) interval duration. (DOC) [file pone.0017584.s001.doc]

| **Table S1.** Adjusted difference (95%CI) in Bazett’s equation-corrected QT (QTb) interval duration | | | |
| --- | --- | --- | --- |
|  | **Mean QTb** | **Difference in QTb** | |
|  |  | **Model 1 ^a^** | **Model 4 ^b^** |
|  |  |  |  |
| **Coffee (cups/day)** |  |  |  |
| 0 | 431.5 (430.2, 432.7) | 0.0 (reference) | 0.0 (reference) |
| <1 | 428.8 (427.3, 430.2) | -0.8 (-2.1, 0.5) | -0.9 (-2.3, 0.4) |
| 1-3 | 427.7 (426.0, 429.3) | -1.9 (-3.6, -0.3) | -1.7 (-3.5, 0.1) |
| 4-5 | 428.4 (425.4, 431.3) | -0.2 (-2.8, 2.4) | 0.8 (-2.0, 3.5) |
| ≥6 | 425.7 (421.0, 430.4) | -1.0 (-4.5, 2.5) | -1.0 (-4.4, 2.4) |
| p-trend | 0.004 | 0.45 | 0.67 |
| **Tea (cups/day)** |  |  |  |
| 0 | 429.1 (427.9, 430.3) | 0.0 (reference) | 0.0 (reference) |
| <1 | 429.4 (427.9, 431.0) | -0.3 (-1.5, 1.0) | 0.0 (-1.4, 1.3) |
| 1-3 | 428.4 (424.7, 432.0) | -1.6 (-4.5, 1.2) | -2.2 (-5.2, 0.8) |
| 4-5 | 428.2 (422.9, 433.5) | -0.2 (-5.1, 4.7) | 0.6 (-4.5, 5.7) |
| ≥6 | 431.9 (421.2, 442.6) | 0.2 (-9.9, 10.4) | -2.3 (-12.2, 7.6) |
| p-trend | 0.90 | 0.49 | 0.30 |
| **Caffeine (mg/day)** |  |  |  |
| <24.2 | 430.9 (429.7, 432.2) | 0.0 (reference) | 0.0 (reference) |
| 24.2-135.2 | 428.1 (426.7, 429.6) | -1.6 (-3.1, -0.1) | -1.3 (-2.9, 0.3) |
| 135.2-274.9 | 431.1 (428.7, 433.5) | -0.1 (-1.5, 1.3) | 0.0 (-1.6, 1.5) |
| ≥274.9 | 427.6 (426.1, 429.1) | -1.7 (-3.1, -0.2) | -1.3 (-2.9, 0.4) |
| p-trend | <0.001 | 0.05 | 0.19 |
| **Smoking** |  |  |  |
| Never | 430.8 (429.5, 432.2) | 0.0 (reference) | 0.0 (reference) |
| Former | 427.7 (426.2, 429.2) | 0.7 (-0.8, 2.2) | 0.6 (-0.8, 1.9) |
| Current | 428.5 (426.8, 430.2) | 0.7 (-1.1, 2.5) | 1.2 (-0.6, 3.1) |
| p-value (Former vs. Never) | <0.001 | 0.38 | 0.44 |
| p-value (Current vs. Never) | 0.01 | 0.46 | 0.19 |
| **Pack-years (current smokers only)** |  |  |  |
| ≤15.6 | 425.7 (423.0, 428.5) | 0.0 (reference) | 0.0 (reference) |
| 15.6-31.5 | 427.1 (424.4, 429.7) | 0.6 (-2.7, 3.8) | 0.4 (-2.8, 3.6) |
| 31.5-49.8 | 429.6 (426.1, 433.2) | 0.8 (-2.3, 3.8) | 0.7 (-2.9, 4.2) |
| ≥49.8 | 431.6 (429.7, 433.6) | 3.2 (0.0, 6.4) | 2.7 (-1.0, 6.4) |
| p-trend | <0.001 | 0.04 | 0.14 |
| **Serum cotinine (ng/ml, current smokers only)** |  |  |  |
| <131.3 | 427.3 (424.5, 430.1) | 0.0 (reference) | 0.0 (reference) |
| 131.3-228.8 | 431.4 (428.6, 434.3) | 2.2 (-1.2, 5.5) | 2.6 (-1.1, 6.3) |
| 228.8-328.8 | 426.7 (424.3, 429.2) | -1.5 (-4.4, 1.5) | -0.8 (-4.2, 2.6) |
| >328.8 | 428.7 (425.4, 431.9) | 0.8 (-2.7, 4.2) | 1.7 (-2.1, 5.5) |
| p-trend | 0.89 | 0.96 | 0.66 |
| **Secondhand smoking (SHS)** |  |  |  |
| SHS unexposed never smokers | 432.2 (429.1, 435.2) | 0.0 (reference) | 0.0 (reference) |
| SHS exposed never smokers | 430.4 (429.1, 431.7) | 0.6 (-2.4, 3.6) | 0.9 (-2.2, 4.0) |
| Current smokers | 428.5 (426.8, 430.2) | 1.2 (-1.9, 4.3) | 2.1 (-1.1, 5.4) |
| p-value (SHS exposed vs.SHS unexposed never smokers) | 0.26 | 0.71 | 0.56 |
| p-value (current smokers vs. SHS unexposed never smokers) | 0.02 | 0.44 | 0.21 |
| **Alcohol (drinks/week)** |  |  |  |
| 0 | 431.6 (430.1, 433.1) | 0.0 (reference) | 0.0 (reference) |
| 1-3 | 426.9 (425.1, 428.7) | -0.4 (-2.0, 1.3) | -0.5 (-2.2, 1.2) |
| 4-6 | 424.6 (421.5, 427.8) | 0.1 (-3.0, 3.3) | 0.3 (-2.8, 3.4) |
| ≥7 | 427.4 (425.5, 429.3) | 1.6 (-0.5, 3.8) | 1.7 (-0.5, 4.0) |
| p-trend | <0.001 | 0.15 | 0.11 |
| **Binge driking** |  |  |  |
| Non-drinker or ex-drinker | 431.4 (430.1, 432.7) | 0.0 (reference) | 0.0 (reference) |
| Current drinker, binge drinking | 426.5 (424.6, 428.3) | 2.2 (0.0, 4.4) ^c^ | 1.6 (-0.6, 3.7) ^c^ |
| p-value ^c^ | <0.001 | 0.06 | 0.17 |
| Current drinker, no binge drinking | 426.3 (424.3, 428.2) | 0.0 (reference) | 0.0 (reference) |
| Current drinker, binge drinking | 426.5 (424.6, 428.3) | 3.1 (0.7, 5.4) ^d^ | 2.0 (-0.4, 4.5) ^d^ |
| p-value ^d^ | 0.87 | 0.01 | 0.11 |
| **Total physical activity (times/week)** |  |  |  |
| 0 | 434.6 (432.9, 436.4) | 0.0 (reference) | 0.0 (reference) |
| 0.1-2.9 | 428.9 (427.6, 430.2) | -0.5 (-2.4, 1.5) | 0.4 (-1.5, 2.2) |
| 3.0-7.9 | 427.3 (425.7, 428.9) | -1.2 (-3.4, 1.0) | -0.8 (-3.0, 1.3) |
| ≥8.0 | 423.9 (421.9, 426.0) | -2.1 (-4.2, -0.1) | -0.8 (-3.1, 1.5) |
| p-trend | <0.001 | 0.04 | 0.24 |
| **Vigorous physical activity (times/week)** |  |  |  |
| 0 | 430.3 (429.3, 431.3) | 0.0 (reference) | 0.0 (reference) |
| 0.1-1.0 | 427.0 (425.0, 429.0) | -1.6 (-3.4, 0.1) | -0.7 (-2.7, 1.3) |
| 1.1-4.0 | 426.4 (423.6, 429.3) | -0.7 (-2.8, 1.4) | -0.2 (-2.5, 2.1) |
| >4 | 428.9 (426.7, 431.0) | -0.7 (-3.1, 1.6) | -0.2 (-2.9, 2.5) |
| p-trend | 0.11 | 0.59 | 0.93 |
|  |  |  |  |
| a. Model 1: Adjusted for age (continuous), race-ethnicity (non-Hispanic white, non-Hispanic black, Mexican-American, other), sex, and RR-interval (restricted quadratic splines with knots at the 5th, 50th, and 95th percentiles) | | | |
| b. Model 4: Fully adjusted models (refer to table 2-5 for details) | | | |
| c. Current drinker, binge drinking vs. non-driker or ex- drinker | | | |
| d. Current drinker, binge drinking vs. current drinker, no binge drinking | | | |
