# Supplementary material for: Coffee, Alcohol, Smoking, Physical Activity and QT Interval Duration: Results from the Third National Health and Nutrition Examination Survey
Source: PLoS One. 2011 Feb 28;6(2):e17584. doi: 10.1371/journal.pone.0017584 (PMC3046251; doi:10.1371/journal.pone.0017584)
Supplement: Table S2 — Adjusted difference (95%CI) in QT interval by sex. (DOC) [file pone.0017584.s002.doc]

| **Table S2.** Adjusted difference (95%CI) in QT interval by sex | | |  | | |
| --- | --- | --- | --- | --- | --- |
|  | **Men** | |  | **Women** | |
|  | **Model 1 ^a^** | **Model 4 ^b^** |  | **Model 1 ^a^** | **Model 4 ^b^** |
|  |  |  |  |  |  |
| **Coffee (cups/day)** |  |  |  |  |  |
| 0 | 0.0 (reference) | 0.0 (reference) |  | 0.0 (reference) | 0.0 (reference) |
| <1 | -1.3 (-3.9, 1.4) | -0.9 (-3.6, 1.7) |  | -0.4 (-2.2, 1.3) | -0.9 (-2.7, 1.0) |
| 1-3 | -1.6 (-4.5, 1.2) | -0.3 (-3.4, 2.7) |  | -2.0 (-4.4, 0.4) | -2.5 (-5.2, 0.3) |
| 4-5 | 0.0 (-4.2, 4.2) | 1.4 (-3.0, 5.8) |  | -0.6 (-3.5, 2.2) | 0.0 (-3.2, 3.2) |
| ≥6 | -2.1 (-6.9, 2.6) | -2.4 (-7.1, 2.4) |  | 0.2 (-4.4, 4.7) | 0.2 (-4.9, 5.3) |
| p-trend | 0.54 | 0.64 |  | 0.61 | 0.75 |
| **Tea (cups/day)** |  |  |  |  |  |
| 0 | 0.0 (reference) | 0.0 (reference) |  | 0.0 (reference) | 0.0 (reference) |
| <1 | -1.5 (-3.3, 0.4) | -0.9 (-2.8, 1.0) |  | 0.5 (-1.0, 2.0) | 0.4 (-1.0, 1.9) |
| 1-3 | -3.4 (-7.8, 1.0) | -3.3 (-8.0, 1.4) |  | -0.6 (-4.1, 2.8) | -1.7 (-5.2, 1.8) |
| 4-5 | 0.9 (-7.6, 9.4) | 1.1 (-7.4, 9.6) |  | -0.4 (-4.2, 3.3) | 0.4 (-3.6, 4.4) |
| ≥6 | 0.3 (-10.3, 10.9) | 1.9 (-8.4, 12.2) |  | 0.6 (-11.5, 12.6) | -3.1 (-17.1, 11.0) |
| p-trend | 0.36 | 0.50 |  | 0.85 | 0.45 |
| **Caffeine (mg/day)** |  |  |  |  |  |
| <24.2 | 0.0 (reference) | 0.0 (reference) |  | 0.0 (reference) | 0.0 (reference) |
| 24.2-135.2 | -1.3 (-3.9, 1.2) | -0.7 (-3.3, 1.9) |  | -1.8 (-4.1, 0.6) | -1.8 (-4.2, 0.7) |
| 135.2-274.9 | -1.3 (-4.5, 1.8) | -1.1 (-4.4, 2.2) |  | 0.7 (-1.2, 2.7) | 0.7 (-1.4, 2.8) |
| ≥274.9 | -1.3 (-4.4, 1.8) | -0.3 (-3.5, 2.9) |  | -1.8 (-3.6, -0.1) | -2.1 (-4.3, 0.2) |
| p-trend | 0.55 | 0.95 |  | 0.05 | 0.08 |
| **Smoking** |  |  |  |  |  |
| Never | 0.0 (reference) | 0.0 (reference) |  | 0.0 (reference) | 0.0 (reference) |
| Former | -0.1 (-2.0, 1.8) | -0.1 (-1.9, 1.8) |  | 0.9 (-1.3, 3.0) | 0.4 (-1.6, 2.5) |
| Current | 0.0 (-2.3, 2.2) | -0.3 (-2.6, 1.9) |  | 1.2 (-0.9, 3.2) | 2.7 (0.4, 4.9) |
| p-value (former vs. never) | 0.93 | 0.96 |  | 0.44 | 0.67 |
| p-value (current vs. never) | 0.97 | 0.78 |  | 0.27 | 0.02 |
| **Pack-years (current smokers only)** |  |  |  |  |  |
| ≤14.9 | 0.0 (reference) | 0.0 (reference) |  | 0.0 (reference) | 0.0 (reference) |
| 14.9-31.4 | -0.5 (-4.5, 3.5) | 0.5 (-3.8, 4.7) |  | 1.3 (-3.5, 6.1) | 0.3 (-4.4, 5.0) |
| 31.4-49.7 | -0.4 (-4.9, 4.1) | -0.2 (-5.4, 5.1) |  | 1.7 (-2.8, 6.1) | 1.8 (-2.5, 6.0) |
| ≥49.7 | 3.0 (-1.4, 7.4) | 3.9 (-1.2, 9.0) |  | 2.8 (-1.3, 6.9) | 0.4 (-4.6, 5.5) |
| p-trend | 0.17 | 0.14 |  | 0.15 | 0.75 |
| **Serum cotinine (ng/ml, current smokers only)** |  |  |  |  |  |
| <127.7 | 0.0 (reference) | 0.0 (reference) |  | 0.0 (reference) | 0.0 (reference) |
| 127.7-226.8 | 2.7 (-1.7, 7.2) | 4.1 (-0.8, 8.9) |  | 1.6 (-3.4, 6.6) | 1.5 (-3.8, 6.8) |
| 226.8-327.6 | -1.8 (-5.4, 1.8) | -0.9 (-4.7, 2.9) |  | -0.4 (-4.6, 3.7) | 0.5 (-4.4, 5.3) |
| >327.6 | 0.7 (-3.3, 4.8) | 2.6 (-1.7, 6.9) |  | 0.7 (-4.1, 5.5) | 0.3 (-4.6, 5.2) |
| p-trend | 0.89 | 0.56 |  | 0.91 | 0.98 |
| **Secondhand smoking (SHS)** |  |  |  |  |  |
| SHS unexposed never smokers | 0.0 (reference) | 0.0 (reference) |  | 0.0 (reference) | 0.0 (reference) |
| SHS exposed never smokers | 1.3 (-2.6, 5.1) | 2.7 (-1.3, 6.7) |  | 0.3 (-3.2, 3.8) | 0.2 (-3.2, 3.6) |
| Current smokers | 1.2 (-2.7, 5.1) | 2.3 (-1.7, 6.3) |  | 1.4 (-2.3, 5.1) | 2.7 (-0.8, 6.2) |
| p-value (SHS exposed vs.SHS unexposed never smokers) | 0.51 | 0.19 |  | 0.86 | 0.90 |
| p-value (Current smokers vs. SHS unexposed never smokers) | 0.55 | 0.26 |  | 0.46 | 0.14 |
| **Alcohol (drinks/week)** |  |  |  |  |  |
| 0 | 0.0 (reference) | 0.0 (reference) |  | 0.0 (reference) | 0.0 (reference) |
| 1-3 | 0.8 (-1.4, 2.9) | 0.5 (-1.8, 2.9) |  | -1.1 (-3.2, 1.1) | -1.0 (-3.2, 1.2) |
| 4-6 | 2.3 (-1.1, 5.8) | 1.5 (-2.2, 5.2) |  | -2.3 (-6.1, 1.4) | -1.2 (-4.7, 2.4) |
| ≥7 | 3.2 (0.3, 6.1) | 1.9 (-1.1, 4.9) |  | -0.4 (-3.6, 2.9) | 1.2 (-2.1, 4.5) |
| p-trend | 0.04 | 0.20 |  | 0.63 | 0.50 |
| **Binge driking** |  |  |  |  |  |
| Non-drinker or ex-drinker | 0.0 (reference) | 0.0 (reference) |  | 0.0 (reference) | 0.0 (reference) |
| Current drinker, binge drinking | 2.8 (0.4, 5.3) ^c^ | 2.0 (-0.4, 4.4) ^c^ |  | 1.1 (-2.9, 5.2) ^c^ | 0.6 (-3.8, 5.0) ^c^ |
| p-value ^c^ | 0.03 | 0.11 |  | 0.58 | 0.79 |
|  |  |  |  |  |  |
| Current drinker, no binge drinking | 0.0 (reference) | 0.0 (reference) |  | 0.0 (reference) | 0.0 (reference) |
| Current drinker, binge drinking | 4.0 (1.6, 6.4) ^d^ | 3.1 (0.6, 5.6) ^d^ |  | 1.7 (-2.3, 5.7) ^d^ | 0.5 (-3.6, 4.5) ^d^ |
| p-value ^d^ | 0.002 | 0.02 |  | 0.42 | 0.82 |
| **Total physical activity (times/week)** |  |  |  |  |  |
| 0 | 0.0 (reference) | 0.0 (reference) |  | 0.0 (reference) | 0.0 (reference) |
| 0.1-2.9 | -0.2 (-2.9, 2.5) | 1.5 (-1.1, 4.2) |  | -0.3 (-2.4, 1.9) | 0.1 (-2.0, 2.2) |
| 3.0-7.9 | 0.4 (-2.4, 3.1) | 1.2 (-1.6, 4.0) |  | -2.2 (-5.0, 0.6) | -2.0 (-4.9, 0.8) |
| ≥8.0 | -0.9 (-4.0, 2.1) | 1.3 (-2.0, 4.5) |  | -3.4 (-6.3, -0.4) | -2.8 (-6.1, 0.6) |
| p-trend | 0.70 | 0.83 |  | 0.01 | 0.03 |
| **Vigorous physical activity (times/week)** |  |  |  |  |  |
| 0 | 0.0 (reference) | 0.0 (reference) |  | 0.0 (reference) | 0.0 (reference) |
| 0.1-1.0 | 0.7 (-2.2, 3.7) | 1.3 (-1.7, 4.3) |  | -3.7 (-6.1, -1.2) | -2.3 (-4.8, 0.2) |
| 1.1-4.0 | -0.9 (-3.5, 1.7) | -0.7 (-3.5, 2.1) |  | -0.4 (-2.8, 1.9) | 0.0 (-2.8, 2.8) |
| >4 | 1.7 (-1.3, 4.7) | 2.8 (-0.8, 6.3) |  | -3.1 (-6.3, 0.1) | -3.0 (-6.6, 0.6) |
| p-trend | 0.31 | 0.16 |  | 0.10 | 0.15 |
|  |  |  |  |  |  |
| a. Model 1: Adjusted for age (continuous), race-ethnicity (non-Hispanic white, non-Hispanic black, Mexican-American, other), sex, and RR-interval (restricted quadratic splines with knots at the 5th, 50th, and 95th percentiles) | | | | | |
| b. Model 4: Fully adjusted models (refer to table 2-5 for details) | | | | | |
| c. Current drinker, binge drinking vs. Non- or ex- drinker | | | | | |
| d. Current drinker, binge drinking vs. Current drinker, no binge drinking | | | | | |
